# Consensus Head Acceleration Measurement Practices (CHAMP): Origins, Methods, Transparency and Disclosure

**DOI:** 10.1007/s10439-022-03025-9

**Published:** 2022-08-03

**Authors:** Kristy B. Arbogast, Jaclyn B. Caccese, Thomas A. Buckley, Andrew S. McIntosh, Kyvory Henderson, Brian D. Stemper, Gary Solomon, Steven P. Broglio, James R. Funk, Jeff R. Crandall

**Affiliations:** 1 Center for Injury Research and Prevention, Children’s Hospital of Philadelphia, 2716 South St., PA 19146 Philadelphia, USA; 2 Department of Pediatrics, University of Pennsylvania, PA Philadelphia, USA; 3 The Ohio State University Chronic Brain Injury Program, The Ohio State University College of Medicine, Columbus, OH USA; 4 Department of Kinesiology and Applied Physiology, University of Delaware, Newark, DE USA; 5 McIntosh Consultancy and Research, Sydney, NSW Australia; 6 Monash University Accident Research Centre, Monash University, Melbourne, VIC Australia; 7 School of Engineering, Edith Cowan University, Perth, WA Australia; 8 Diversified Technical Systems, Inc., Seal Beach, CA USA; 9 Joint Department of Biomedical Engineering, Medical College of Wisconsin & Marquette University, Milwaukee, WI USA; 10 Department of Neurosurgery, Medical College of Wisconsin, Milwaukee, WI USA; 11 Neuroscience Research, Zablocki Veterans Affairs Medical Center, Milwaukee, WI USA; 12 National Football League Player Health and Safety, New York, NY USA; 13 Michigan Concussion Center, University of Michigan, Ann Arbor, MI USA; 14 Biocore LLC, Charlottesville, VA USA

**Keywords:** Head kinematics, Best practices, Sensors, Sport safety, Sport-related concussion

## Abstract

The use of head kinematic measurement devices has recently proliferated owing to technology advances that make such measurement more feasible. In parallel, demand to understand the biomechanics of head impacts and injury in sports and the military has increased as the burden of such loading on the brain has received focused attention. As a result, the field has matured to the point of needing methodological guidelines to improve the rigor and consistency of research and reduce the risk of scientific bias. To this end, a diverse group of scientists undertook a comprehensive effort to define current best practices in head kinematic measurement, culminating in a series of manuscripts outlining consensus methodologies and companion summary statements. Summary statements were discussed, revised, and voted upon at the Consensus Head Acceleration Measurement Practices (CHAMP) Conference in March 2022. This manuscript summarizes the motivation and methods of the consensus process and introduces recommended reporting checklists to be used to increase transparency and rigor of future experimental design and publication of work in this field. The checklists provide an accessible means for researchers to apply the best practices summarized in the companion manuscripts when reporting studies utilizing head kinematic measurement in sport and military settings.

**Supplementary Information:**

The online version contains supplementary material available at 10.1007/s10439-022-03025-9.

## Introduction

Accelerometers were first implemented in studies of American football athletes to elucidate head impact biomechanics associated with sport-related concussion (SRC) in 1965.^34^ While initial designs were rudimentary by today’s standards, advances in technology (e.g., wireless communication, smaller electronics, gyroscopes, etc.) have resulted in helmet,^3,9^ mouthguard,^16^ ear,^29^ and epidermal^38^ mounted systems (see O’Connor *et al*.^28^ for review). Parallel to these advances have been the implementation of video analyses,^2,23,27^ rigid body reconstructions,^24^ dummy reconstructions,^13,32^ computer modeling,^4,18,31^ and increasingly sophisticated data analytics,^10,44^ paired with head acceleration measurement devices to quantify head impact exposure in sports. The result has been increased research to better understand the head kinematics of players participating in American football,^6,11,14,20,36^ football/soccer,^19,21^ ice hockey,^12,25,43^ rugby,^17^ and other sports.^16,22,29^ This research has also been extended to focus on blunt head impacts experienced during military training.^35^ Together, these studies have contributed to the body of literature that has moved concussion science forward. As the use of devices to measure head kinematics proliferates,^30^ the field is ready for guidelines pertaining to methodological rigor to improve the consistency of research and reduce the risk of scientific bias.

## Consensus Head Acceleration Measurement Practices (CHAMP) Group Origins and Methods

The Consensus Head Acceleration Measurement Practices (CHAMP) group was founded to develop and recommend best practices for the collecting, analyzing, and reporting of head acceleration measurement data in sport. A leadership group (identified in the supplementary materials) formed and identified six areas of focus:

- Study Design and Statistical Analysis in Studies of Head Acceleration Measurement
- Laboratory Validation of Wearable Head Kinematic Devices
- On-Field Deployment and Validation of Wearable Head Kinematic Devices
- Video Analysis of Head Acceleration Events
- Physical Reconstruction of Head Acceleration Events
- Computational Modeling of Head Acceleration Events

The leadership group approached experts in the field to lead each section, and those team leaders were asked to recruit members to their working group, encouraging broad participation throughout the field. These teams of experts drafted documents (“chapters”) that outline currently recommended best practices for many aspects of head acceleration measurement—these are summarized by the other manuscripts in this series.

The workgroups, as well as a broader group of key stakeholders, convened at a consensus conference held in Philadelphia, Pennsylvania, USA on March 24–25, 2022. At the conference (held both in person and *via* a virtual option), workgroup leaders presented their work providing details and support for a set of consensus statements (5–7 statements for each “chapter”). All attendees at the conference participated in an open scientific discussion of the key concepts and then formally voted on each consensus statement. *A priori*, the following criteria were established for the voting procedures. Eighty percent or greater agreement with each statement would be required for a statement to be accepted with no further discussion. Less than 20% agreement with a statement would result in the statement being removed from consideration. Initial support between 20 and 80% for a statement would result in further discussion and revision of the statement, striving for at least 80% agreement. If 80% agreement could not be reached, a group of those that dissented would be identified and asked to draft a brief counterpoint to the statement. Of note, at least 80% agreement was reached for all statements, often with detailed discussion and revision; thus, no dissension statements were necessary. A summary of the voting results is contained in the Supplementary materials.

## Transparency and Disclosure *via* Reporting Checklists

In addition to the scientific topics covered in the companion manuscripts in this series, an additional area of focus was identified: transparency and disclosure. Systematic and transparent disclosure of detailed methods used in head acceleration measurement studies is key to improved rigor and reproducibility of research in this area. Further, transparency around research conducted in partnership with industry sponsors and the use of proprietary algorithms to process head kinematic data from wearable devices is important to disclose. Transparency and disclosure can be achieved by identifying critical elements that require reporting. Suboptimal reporting of these elements allows authors, intentionally or inadvertently, to avoid highlighting any weakness in the methodological aspects of their studies. Other fields of scientific inquiry have enhanced transparency and disclosure through the development of checklists outlining reporting recommendations.^5,7,42^ Led by efforts such as CONSORT (www.consort-statement.org) and EQUATOR (www.equator-network.org), a series of reporting checklists exist for numerous study designs^1,26,37,39,40^ and their use has been shown to positively contribute to completeness and transparency of published work.^8,15,33,41^

The CHAMP Transparency and Disclosure working group adopted this approach and developed checklists that align with five of the technical manuscripts that are associated with a particular methodologic approach (Lab Validation, On-field Deployment and Validation, Video Reconstruction, Physical Reconstruction, and Computer Modeling) (Tables 1, 2, 3, 4, 5). These checklists were developed to assist authors in reporting the conditions under which studies utilizing head kinematic measurement were designed and implemented. The checklist elements also provide guidance to journal editors and manuscript reviewers on key components to consider when evaluating the scientific merit of the work and give structure to readers as they review the literature. Checklists are not intended to specify a particular study design, analytical method, or reporting format, nor are they intended to replace existing study reporting checklists like CONSORT that may apply to a given study. Rather, they outline the elements that are necessary for transparent reporting. For each item in each checklist, we include a brief explanation and elaboration of the items, along with examples of transparent reporting and disclosure in the existing literature. Not all items have existing examples to highlight.

**Table 1:** CHAMP 2022 checklist of information to include when reporting laboratory validation studies of head acceleration measurement devices.

Checklist Item	Explanation	Example(s)	Reported on Page No
1. Sensor Technology and Specifications			
(1a) Device model name	The name or model of device used to collect data	PMID: 33051745, “The Cue, GForceTracker, and Shockbox sensors were mounted directly inside the helmet.”	________
(1b) Sensor type	The type of sensor (e.g., triaxial linear accelerometer, triaxial ARS)	PMID: 26268586, “The X2 system has a 3-axis linear accelerometer and a 3-axis angular rate sensor…”	________
(1d) Sensor sample rate	The sampling rate of the sensor	PMID: 32975553, “The sensor records 62 ms of data at 1000 Hz….”	________
(1e) Sensor magnitude range	The range of magnitudes the sensor can record	PMID: 23604848, “Mouthguard sensing is accomplished via a triaxial accelerometer (ADXL377, Analog Devices, Inc., Norwood, MA, USA) with 200 g maximum per axis and a tri-axial angular rate gyroscope (L3G4200D, ST Microelectronics, Geneva, Switzerland) with 40 rad/s maximum per axis.”	________
(1f) If applicable, device hardware/firmware version number	The version number related to the hardware/firmware for the device	PMID: 29613824, “Hardware and firmware were fully up to date according to the manufacturers at the time of testing [xPatch: Hardware updated Oct 2014, Software and firmware updated Aug 2017; SIM-G: Hardware updated Jun 2014, Software and firmware updated Aug 2017]”	________
(1g) Recording trigger threshold	The sensing threshold (e.g., 10 g) for an event to be recorded on the head acceleration measurement device and how the trigger threshold is evaluated	PMID: 23891566, “The helmet recorded …if the impact exceeded 10 g.”	________
(1h) Pre-trigger duration	Duration of pre-trigger data recorded	PMID: 32975553, “…10 ms before and 52 ms after linear acceleration exceeds the threshold.”	________
(1i) Post-trigger duration	Duration of post-trigger data recorded	PMID: 32975553, “…10 ms before and 52 ms after linear acceleration exceeds the threshold.”	________
(1j) Device form factor and attachment	The type of device/how the device is mounted (e.g., mouthguard)	PMID: 30802147, “MV1 (MVTrak) is a sensor system designed for custom-molded placement in the left external ear canal to optimize coupling to the head.”	________
2. Surrogate Selection			
(2a) Surrogate used	The surrogate used (e.g., non-biofidelic [by intent] test device, anthropometric test device (ATD), post-mortem human subjects (PMHS), human volunteers)	PMID: 23846161, “A Hybrid III (HIII) 50th percentile male ATD head and neck with the 3-2-2-2 accelerometer array was rigidly mounted at T1.”	________
(2b) Inertial properties of surrogate	Geometry and mass—including what reference population is intended to be represented by this surrogate	PMID: 27155744, “This ATD had the inertial properties of a 50th percentile male head.”	________
(2c) If applicable, modifications made to standard surrogates	Any modifications made to standard surrogates for this study	PMID: 34263384, “Modifications to the NOCSAE headform include a mouth cut-out for mounting dentitions and a Hybrid-III neck adapter to replace the standard rigid neck and allow 6DOF head motion.”	________
(2d) If applicable, corresponding neckform and/or other body segments used	The neckform used to simulate head-neck response (e.g., Hybrid III neck, THOR neck) and/or other body segments (e.g., torso) used to simulate the system mass	PMID: 21994068, “… mounted to a standard HIII neck was used to replicate the response of a football player’s head. Per manufacturer’s specification, the cable in the HIII neck was tensioned to 1.1 Nm (10 in·lb.).”	________
(2e) Modifications made to standard neckforms, if applicable	Any modifications made to standard neckforms for this particular study	PMID: 33000448, “…the lower neck mount of the Hybrid III dummy was modified to incorporate a spherical ball joint that allowed for lateral flexion and twist of the neck.”	________
(2f) Validation of the surrogate	Evidence that the surrogate has been shown to produce a validated response for the chosen application	PMID: 29613824, “These reference sensors have been found to exhibit high fidelity (ref) and were considered to quantify the true head kinematics of the headform during impact.”	________
(2g) Mounting of the device on the surrogate	Details on how the device is mounted on the surrogate and the biofidelity of that mounting	PMID: 29383374, “The dental model was rigidly attached to the ATD headform in the place of the upper dentition, and the instrumented mouthpiece was mounted on the dental model, with the lower jaw firmly clamped to the mouthguard simulating jaw clenching…”	________
(2h) Factors related to coupling of the device to the surrogate	Specific parameters that could influence coupling of the device to the surrogate (e.g., helmet fit, skin/hair surrogate, use of nylon skull cap, sweat, jaw mechanics)	PMID: 23891566, “The helmet was fit by inflating the Z-pad bladders until they contacted the head.”	________
3. Test Conditions			
(3a) Test device	The device used in testing (linear impactor, pendulum, drop tower)	PMID: 21451177, “The helmet was impacted using a pneumatic linear impactor.”	________
(3b) Impactor surface and mass	The type and material of the impact interface (elastomer padding, use of anvils, etc.). Provide the mass and its relevance to desired test conditions	PMID: 24920257, “The impactor mass was 14 kg and was padded with a 36 mm thick, 127 mm diameter vinyl nitrile pad (Impax VN 600, Der-Tex Corp, Saco, ME) without the standard hard plastic cap. This configuration generated an impact amplitude and duration similar to that observed during helmet-to-helmet impacts.”	________
(3c) Surrogate orientation and mounting, if applicable	How the surrogate was placed in the test device	PMID: 24920257, “Both headforms were mounted on a 50th percentile male Hybrid III neck mounted to a table free to slide horizontally parallel to the impactor’s axis.”	________
(3d) Impact velocity	The velocities used in testing and their relevance to desired test conditions	PMID: 32989591, “Regarding the impact velocities used for the testing, three of the used velocities (5.5, 7.4, and 9.3 m/s) are based on the National Football League (NFL) helmet test protocol, and an additional lower velocity (3.6 m/s) was added to analyze impacts of lower intensity as well.”	________
(3e) Impact duration	The duration(s) used in testing and their relevance to desired test conditions	PMID: 29613824, “The helmeted tests yielded average impact durations of 10.7 (1.3) milliseconds. The padded impactor to bare head condition was performed with a vinyl-nitrile foam impactor face measuring 127 mm in diameter and 40 mm thick. These tests yielded average impact durations of 12.5 (1.3) milliseconds and were chosen to provide similar impacts to the helmeted condition without the effect of the helmet. The rigid impactor to bare head condition was performed with the same flat, rigid, nylon impactor face from the helmeted tests to be representative of impact magnitudes and durations seen in unhelmeted impacts. These impacts yielded average durations of 3.6 (0.25) milliseconds.”	________
(3f) Impact location	The impact location(s) used in testing and their relevance to desired test conditions	PMID: 29613824, “Impacts were performed to the front, front boss, rear boss, and rear locations of the headform at targeted linear acceleration magnitudes of 25, 50, 75, and 100 g. Impact locations were equally spaced around the head and chosen because of their variability in direction of force.”	________
(3g) Impact direction	The direction(s) of impact used in testing and their relevance to desired test conditions	PMID: 26268586, “Ten impacts were nominally centroidal, i.e., the impactor’s axis passed near a vertical axis through the headform’s COG.” PMID: 26268586, “The front-oblique test condition was intended to represent a centric impact (head CG path eccentricity = 65 mm) and the rear eccentric test condition was intended as a more eccentric impact (head CG path eccentricity = 101 mm).”	________
(3h) Number of trials	The number of trials performed for each of the test conditions	PMID: 17597937, “Three drops were performed at each location.”	________
(3i) If applicable, helmet manufacturer/model name	The name of the manufacturer/model of the helmet used in impact testing	PMID: 29613824, A large Riddell Speed (Riddell, Elyria, OH) football helmet without the facemask was worn by the headform throughout helmeted tests	________
(3j) Repeatability and reproducibility of test conditions	Methods used to evaluate the repeatability and reproducibility of the test conditions and surrogate	PMID: 24920257, “Repeatability was assessed using the COV, which equals the ratio of the standard deviation (SD) to the mean, expressed as a percentage. Repeatability was categorized as excellent (COV ≤ 3%), acceptable (3 < COV ≤ 7%), marginal (7 < COV ≤ 10%) and poor (COV > 10%). The COVs for PLA and PAA were calculated for each series of five repeated tests for all eight impact conditions in each lab.”	________
4. Reference Sensor Measurement			
(4a) Reference sensor type and model	The type of sensor or measurement device used as a reference (triaxial accelerometer, nine-accelerometer package, high-speed video), including the sensor part number	PMID: 26268586, “…a 3-2-2-2 array of linear accelerometers (Endevco 7264B-2000 g, San Juan Capistrano, CA)”	________
(4b) Reference sensor mounting	The method and location for reference sensor mounting	PMID: 26268586, “…a 3-2-2-2 array of linear accelerometers (Endevco 7264B-2000 g, San Juan Capistrano, CA) installed in a compact cluster (*rx* = *ry* = 34 mm, *rz* = 27 mm) in a modified load-sensing headform (MLSH) based on the 50th percentile male Hybrid III headform.”	________
(4c) Reference sensor sampling rate	The sampling rate of the reference sensor	PMID: 26268586, “…modified load-sensing headform (MLSH) based on the 50th percentile male Hybrid III headform. Accelerometer data were acquired at 10 kHz with hardware anti-aliasing filters prior to digitization (SAE Channel Class 1000).”	________
(4d) Reference sensor magnitude range	The range of magnitudes the reference sensor can record		________
(4e) Reference sensor filtering	Filtering methods used for the reference measurements	PMID: 29613824, “The reference data were filtered at CFC 1000 for linear acceleration and CFC 155 for rotational velocity.”	________
(4f) Time synching of reference sensor to head kinematic device	Method for synching reference data to wearable device data	PMID: 31297724, “Mouthpiece and reference traces were time-aligned such that the first data point that crossed the 5 g trigger threshold was set to time *t* = 0.”	________
5. Advanced Post-processing			
(5a) Data transformation	Methods used to transform recorded data to analyzable data (e.g., numerical integration from angular velocity to angular acceleration, transformation from the location of the sensor to the center of gravity of the head, if transformation used, specify measurements defining the location to which data is transformed, must disclose if a “black box” algorithm was used)	PMID: 31122140, “The acceleration data are transformed to calculate linear acceleration at the centre of gravity of the head. Rotational acceleration is calculated from rotational velocity using five-point differentiation. Both the transformation and differentiation were carried using the software supplied by X2Biosystems.” PMID: 34263384, “Kinematics measured by the mouthpiece were transformed to a local head coordinate system using a rigid body transformation based on the geometry of each headform. Detailed 3D surface scans of both headforms with the IM affixed to the upper dentition were obtained to determine the location and orientation of the sensing elements in relation to the head CG (Artec Eva, Artec 3D, Santa Clara, CA). Reference measurements at the maxilla and device measurements from the electronics board inside the head of the MLSH were transformed to the head CG based on detailed computer drawings”	________
(5b) Kinematic data filtering	Any filtering used for processing data collected from a wearable device; must disclose if manufacturer “black box” post-processing was used. Include offset removal	PMID: 29383374, “Raw signals were low-pass filtered according to Society of Automotive Engineers protocols. The mouthpiece data used threshold frequencies of 300 Hz and 110 Hz for linear acceleration and angular velocity, respectively, with 110 Hz being the bandwidth limit for the gyroscope.” PMID: 30802147, “Raw data are uploaded to the MVTrak server before being processed by the producer’s algorithm.”	________
(5c) Other post-processing techniques	Any software or hardware used for processing data collected from a wearable device (e.g., impact detection filtering, infrared system); must disclose if manufacturer “black box” post-processing was used. Provide details on validation of post-processing techniques (e.g., training data set used)	10.1177/1754337117739458, “…data were processed using proprietary algorithms from which the resultant peak linear acceleration (PLA) and peak angular acceleration (PAA) impact magnitude measures were output.”	________
(5d) Event removal	Clear, objective methods for sensor event removal, if any sensor events are removed from analysis	PMID: 32975553, “A positive single axis maximum of 28.9 rad/s and negative single axis absolute maximum of 29.1 rad/s were determined. One trial was removed from analysis because this maximum angular velocity measurement was sustained for more than five consecutive data points.”	________
6. Analytical Methods and Data Reporting			
(6a) Validation metrics, including equations used to derive metrics, if applicable	Description of each primary and secondary validation metric (e.g., impact counts, peak linear acceleration, change in angular velocity)	PMID: 32975553, “Key event characteristics include peak angular velocity (i.e., maximum velocity during event), rise time (i.e., time for angular velocity to reach peak velocity from event start at velocity surpassing 5% of peak), fall time (i.e., time from peak velocity to 5% of the peak), and a proxy for average angular acceleration (i.e., approximated by taking the ratio of peak angular velocity and the rise time).”	________
(6b) Statistical and analytical methods for comparison	The statistical and analytical methods used to compare the wearable device data to the reference measures (e.g., recall, RMS error, general linear mixed models)	PMID: 31297724, “Average resultant peak percent error was used to determine agreement between reference data and the mouthpiece data. Normalized root-mean-square error (NRMS) was used to determine agreement over the entire impact duration recorded by the mouthpiece (60 ms).”	________

**Table 2:** CHAMP 2022 checklist of information to include when reporting studies of on-field deployment and validation of wearable head acceleration measurement devices.

Checklist item	Explanation	Example(s)	Reported on Page No
1. Sensor Technology and Specifications			
(1a) Device model name	The name or model of device used to collect data	PMID: 15654184, “This study used the Head Impact Telemetry (HIT) System (Simbex, Lebanon, NH)…consists of sensors (6 linear accelerometers and 1 temperature)…”	________
(1b) Sensor type	The type of sensor (e.g., triaxial linear accelerometer, triaxial ARS)	PMID: 339862230, “The SIM-G device comprises a high and low-g triaxial accelerometer for linear acceleration measurement (3 g–150 g) and a triaxial gyroscope for angular velocity measurement”	________
(1c) Sensor sample rate	The sampling rate of the sensor	PMID: 29809079, “The sensor sampled linear accelerations at a rate of 1 kHz and rotational accelerations at 800 Hz.”	________
(1d) Sensor magnitude range	The range of magnitudes the sensor can record	PMID: 339862230, “The SIM-G device comprises a high and low-g triaxial accelerometer for linear acceleration measurement (3 g–150 g) and a triaxial gyroscope for angular velocity measurement”	________
(1e) If applicable, device hardware/firmware version number	The version number related to the hardware/firmware for the device	PMID: 29373056, “The Smart Impact Monitor (SIM; firmware version 3.7; SIM-G, version 3.3; AP, version 0.9.150413; software, Triax Technologies, Norwalk, CT) was used to quantify head acceleration.”	________
(1f) Recording trigger threshold	The sensing threshold (e.g., 10 g) for an event to be recorded on the head acceleration measurement device and how the trigger threshold is evaluated	PMID: 32913379, “The pre-set trigger for the device to record and download an impact was 10 g. Any impacts below this threshold were not recorded.” PMID: 31388849, “Data acquisition triggered any time a single accelerometer exceeded a 9.6-g threshold.”	________
(1g) Pre- and post-trigger duration	Duration of pre-trigger data recorded	PMID: 27598519, “When an impact above the threshold occurred, information regarding 10 ms before and 52 ms after the impact was transmitted…”	________
(1h) Device form factor and attachment	The type of device/how the device is mounted (e.g., mouthguard)	PMID: 33152691, “Each athlete was fit with a custom-fit mouthpiece instrumented to measure linear and rotational head kinematics during on-field impacts,”	________
(1i) If applicable, device fitting procedures	Procedures for fitting wearable device (e.g., custom-formed mouthguard)	PMID: 33152691, “Dental impressions were obtained from each athlete by a trained dental professional, and a dental model was poured from the dental impression. A custom-fit mouthpiece made of acrylic material was created for each athlete.”	________
(1j) If applicable, helmet manufacturer/model name	The name of the manufacturer/model of the helmet worn by participants in helmeted sports	PMID: 32936594, “Eligible participants for the study wore a Riddell Revolution, Speed, or Speed Flex helmet to accommodate a HIT System encoder…”	________
(1k) Evidence of device kinematic validation	Details regarding device kinematic validation that exists in prior literature	PMID: 29321637, “The mouthguard…has approximately 10% error in measuring peak head linear acceleration, angular acceleration, and angular velocity in dummy head validation (ref).”	________
2. On-Field Logistics			
(2a) Participants	Eligibility criteria for participants (e.g., sport, sex/gender, age, level of play), number of participants approached, number of participants enrolled, number of participants that were ultimately excluded (e.g. incomplete data sets, dropped out of study)	PMID: 31240507, “A total of 340 players from six National Collegiate Athletic Association (NCAA) football programs (two of which were military service academies) participated in this study…”	________
(2b) Study settings/location	Settings and locations where the data were collected (e.g., laboratory, field, practice, games, tournaments)	PMID: 30362082, “Head impact data were recorded for all practice, scrimmage and game activities during the 2015, 2016, and 2017 football seasons, including spring practice, preseason training camp, and regular season practice and games.”	________
(2c) Data collection dates	Seasons/years the data were collected	PMID: 30362082, “Head impact data were recorded for all practice, scrimmage and game activities during the 2015, 2016, and 2017 football seasons, including spring practice, preseason training camp, and regular season practice and games.”	________
(2d) Device usage	Actions to verify device usage (e.g., device is functional, battery is charged, device is attached securely and properly)	PMID: 32255667, “All X-Patch devices were tested for basic functionality (eg, battery life) before use…86 players had patches that detached (60%) or became faulty (40%), and HAEs from these patches were excluded from further analysis.”	________
3. Head Acceleration Event Verification			
(3a) Method of impact verification	Method used to confirm device-recorded events were actual head acceleration events (e.g., video or observer, support vector machine classification, proximity sensor); state if none used	PMID: 33738313, “Video review of all sensor-recorded events was used to identify actual head impact events…” PMID: 34549342, “All recorded events were processed by the MiGNet program, a validated, deep learning algorithm that distinguishes true head impacts from false positive events caused by mouthguard handling, application, or other movements that are unrelated to impacts (ref).” PMID: 32130020, “…the proprietary manufacturer software labeled each sensor-recorded event as either a “valid” or “spurious” impact.”	________
(3b) Time synchronization method	Methods used to synchronize video and wearable device clocks (e.g., video recording the machine clock)	PMID: 28541813, “At the start of each game, with the game official’s audible whistle and the start of the game clock, the videographer displayed a visual marker (clock) of the date and start time of each game. Simultaneously, the “dummy” sensor was struck 5 times in full view of the camera, with the first impact being used to synchronize. At the conclusion of each game, the same procedure was repeated to time stamp the end of play.”	________
4. Data Windowing			
(4a) Temporal windowing	Parameters and methods used for time-windowing (e.g., start and end timepoints of session, elimination of scheduled and unscheduled stoppages, based on video confirmation)	PMID: 32130020, “…the timestamp in the video footage was used to determine the time points associated with the start and end of each half, as indicated by the whistle of the referee, and sensor data outside of verified game times were excluded.”	________
(4b) Temporospatial windowing	Parameters and methods used for player-windowing (e.g., timepoints when a positional group or player enters or leaves the playing area during the session, start and end timepoints of an athlete actively participating in the session, based on video confirmation, proximity sensors, global positioning system)	PMID: 26674407, “All accelerations recorded via the helmets were crosschecked to the Team AMS software [GPS data] to investigate when and where the event occurred.”	________
(4c) Kinematic windowing	Parameters and methods used for windowing the events based on kinematic measures	PMID: 31388849, “Any impacts with peak resultant linear acceleration below 10 g were not included in this analysis as they can be associated with non-impact dynamic movements in the athlete… System output files to ensure that all athlete and impact information values were included, filtering out any impacts that exceeded 200 g and 10,000 rad/s2”	
5. Video Verification			
(5a) Type of video review, if applicable	Guided video review (e.g., to confirm true positive events and identify/remove false positive events) and/or blinded video review (e.g., to quantify false negatives)	PMID: 33078368, “In the first stage, a pool of 16 human reviewers were blinded to the sensor event data and identified head impacts occurring to instrumented players on the field (1 reviewer per player).”	________
(5b) Video recording parameters, if applicable	The number and type of video recording devices (e.g., number of cameras, placement, resolution, and frame rate of video recordings)	PMID: 27432843, “A research assistant captured game video by using a professional grade video camera (Panasonic HMC-40, Secaucus, NJ) placed above the press box ∼3 stories high at the 50-yard line. Video was recorded in full high-definition with a resolution of 1080 × 720 at 24 frames per second.”	________
(5c) Video/observer review parameters, if applicable	The number and type of video reviewers or on-field observers (e.g., number of raters, calculations of inter- and/or intra-rater reliability, level of experience)	PMID: 31075762, “Two independent reviewers analyzed the on-field video data using Kinovea (experimental version 0.8.26) video analysis software independent of the biomechanics.”	________
(5d) Contextual information of head impacts	Other head acceleration or head impact event parameters characterized from video/observer (e.g., impact location)	PMID: 32303477, “…the videos were later reviewed by research staff to eliminate false positives, confirm impact locations on the head, and identify impact mechanisms and player positions.”	________
6. Advanced Post-processing			
(6a) Data transformation	Methods used to transform recorded data to analyzable data (e.g., numerical integration from angular velocity to angular acceleration, transformation from the location of the sensor to the center of gravity of the head, must disclose if a “black box” algorithm was used)	PMID: 34689676, “…mouthpiece-recorded data were filtered, zero-offset, rotated to match a conventional coordinate system, and transformed to the athlete’s head centre of gravity using a subject-specific transformation.”	________
(6b) Kinematic data filtering	Any filtering used for processing data collected from a sensor; must disclose if manufacturer “black box” post-processing was used. Include offset removal	PMID: 34463209, “All data were filtered using a 4th order, zero lag, low-pass Butterworth filter to remove high-frequency noise. A single cut-off frequency was not found to be appropriate for all impacts, due to variability in the underlying signal components. Consequently, impact-specific, optimal cut-off frequencies were determined for each impact using residual analysis. Filtering was applied to vector component data.”	________
(6c) Other post-processing techniques	Any software or hardware used for processing data collected from a sensor (e.g., impact detection filtering, infrared system); must disclose if manufacturer “black box” post-processing was used. Provide details on validation of post-processing techniques (e.g., training data set used)	PMID: 29321637, “A two-class SVM [support vector machine] classifier was trained to differentiate head impacts from the nonimpacts.” PMID: 29321637, “Prior to training the classifier, we used infrared (IR) device placement measurements to filter out recordings where the mouthguard was not coupled to the upper jaw. Then, features were extracted from the kinematic sensor measurements to train an SVM classifier that distinguishes between impacts and nonimpacts.”	________
7. Data Reporting			
(7a) Event definition	A head acceleration event is defined as an event/incident that gives rise to an acceleration response of the head caused by an external short-duration collision force applied directly to the head or indirectly via the body in sport, recreational, military, or other activities of interest (e.g., direct or indirect head acceleration events); a head impact event is defined as a contact event involving direct contact to the head (i.e., direct head acceleration event)	PMID: 33183139, “Head acceleration events (HAEs) were monitored using the xPatch.”	________
(7b) Device-recorded events	Number of events reported by the devices before verification and/or processing	PMID: 30802147, “…the MV1 sensors recorded 2039 nominal head impact events…”	________
(7c) Head acceleration events	Number of true positive acceleration events after verification and post-processing	PMID: 33986230, “Female athletes sustained 271 head impacts during 18 games and male athletes sustained 1041 head impacts during 23 games.”	________
(7d) Device performance	False positive rates (and/or false negative rates with blinded review)	PMID: 31000457, “Among the 66 (56.1%) cases with HAEs (PLA ≥ 30 g) that could be verified on video, 48 (72.7%) were true positive for direct or indirect head impact and 18 (27.3%) were false negatives.”	________
(7e) Athlete exposures	If reporting rates, definition of athlete exposure used (e.g., game/practice, minutes of play, season) and how that data was collected	PMID: 25098659, “In men’s ice hockey, head impacts for individual players resulting from contact with another player occurred at a frequency of approximately once in every 2 games (0.46 per game).” PMID: 30579266, “The impact rate for each drill was described as the impacts per player per minute (ppm).”	________
(7f) Outcomes measures, including equations used to derive outcomes, if applicable	Description of each primary and secondary outcome measure (e.g., peak linear acceleration, head impact power)	PMID: 29856659, “Descriptive statistics were calculated for impact counts, locations, and magnitudes in terms of peak linear acceleration (PLA; g), peak angular acceleration (PAA; rad/s2), and HITsp units.”	________
(7g) If relevant, equations used to derive outcomes	The algorithms used to derive each outcome measure	PMID: 23864337, “Risks associated with each head impact for each player were summed to compute the risk weighted cumulative exposure (RWE) for the season.”	________
(7h) If relevant, categories of direction of impact	Definition and method of determining impact location (e.g., recorded as azimuth and elevation with respect to the head CG)	PMID: 21716150, “Impact location, recorded as azimuth and elevation with respect to the head CG, was categorized into five general location bins (Fig. 2): Front (F), Back (B), Left Side (L), Right Side (R), and Top (T).”	________

**Table 3:** CHAMP 2022 checklist of information to include when reporting video reconstruction studies of head acceleration events.

Checklist item	Explanation	Example(s)	Reported on Page No
1. Study Design and Case Selection			
(1a) Study purpose	The purpose of the study shall be explained clearly—e.g. Collect observational data, compare injury to controls, evaluate interventions	PMID: 28632058, “The goal of this study is to assess the accuracy of the MBIM method relative to reflective marker-based motion analysis data for estimating six degree of freedom head displacements and velocities in a staged pedestrian impact scenario at 40 km/h.”	________
(1b) Video source material	The settings and locations of the events and how the video footage was obtained (e.g., recorded for research purposes, game film)	PMID: 30398897, “For each injury play, NFL Films provided all available video footage from 4 distinct video sources in the highest-quality formats available from each source: (1) “all 22” footage shot by the hosting team, which included the wide-angle end zone and sideline views typically used by coaches and teams for game review purposes; (2) network broadcast footage with replays, which included all footage aired during the televised network broadcast of the game; (3) NFL Films footage, which is untelevised footage frequently shot for the purposes of NFL documentaries; and (4) network melt reel footage, which is footage shot by the network but not included in the televised video feed of the game.”	________
(1c) Eligibility criteria for included events	Eligibility criteria for included events (e.g., sport, sex/gender, age, level of play), type of impact, and definition of object being tracked (e.g., player, helmet)	PMID: 30398897, “All reported concussions sustained in an NFL preseason, regular season, or postseason game played during the 2 target seasons were included in this study.”	
2. Physical Camera Specifications			
(2a) Number and type of cameras	Number and type of cameras used for the recreation of the event	PMID: 34274559, “Eleven action cameras (HERO6; GoPro, Inc., San Mateo, CA, USA) with 41° field of view (FOV) lenses recorded video at 2.7 K resolution and 120 frames per second (fps) with a shutter speed of 1/1920s…”	________
(2b) Relative locations of the camera	The location of the camera relative to the event being recorded and how far apart, in linear and angular dimensions, the cameras from each other	PMID: 34274559, “…four cameras along each sideline at 15-yard intervals and three cameras across the back of the end zone.” PMID: 30274537, “The distance between the camera and impact area ranged from 20 to 100 m.” PMID: 30274537, “Angle of separation between camera views ranged from 66° to 96°…”	________
(2c) Camera calibration or alignment	The methods for calibrating cameras and/or aligning camera views	PMID: 32095268, “Common points were selected in the video clips and laser scan data to align the camera views.”	________
(2d) Camera field of view	Field of view of the cameras	PMID: 34274559, “Eleven action cameras (HERO6; GoPro, Inc., San Mateo, CA, USA) with 41° field of view (FOV) lenses recorded video at 2.7 K resolution and 120 frames per second (fps) with a shutter speed of 1/1920s…”	________
(2e) Camera height	Height of the camera relative to the ground and/or location of the event	PMID: 30274537, Ranged from − 1.5 to − 48.5 m (Table 1)	________
(2f) Camera angle of incident	The angle of the camera relative to the ground that results from the camera’s height off the ground	PMID: 14519212, “Cameras 3 and 4 were 31° and 57° from the sideline.”	________
(2g) Landmarks	Landmarks used to recreate the 3D field	10.1007/s12283-018-0263-4, “A calibration grid was placed in Kinovea to cover the entirety of the exercise test area where the speed was being measured… The grid was then assigned the corner-to-corner distances that matched the measurements of those markings on the ice, establishing a calibrated grid.”	________
(2h) Physical obstructions and/or environmental conditions	How were physical obstructions (e.g., structures or people) and/or glare, light levels, reflections managed	10.4271/2018-01-0516, “Other factors like the number of photographs, the specific vantage of the photographs, and occlusion of recognizable features within these photographs can also limit the number of common features available for use in camera matching. In these instances, camera matching solutions can be improved by extending the 3D environment to include objects visible in the distance such as mountains, valleys, and other notable landmarks that are typically outside of the scope of 3D scene mapping.”	________
3. Camera Recording Specifications			
(3a) Frame rate	The rate at which consecutive images or frames are captured or displayed (e.g., in frames per second); must identify if the original video had interlaced frames and the analysis used de-interlaced frames	PMID: 34274559, “Eleven action cameras (HERO6; GoPro, Inc., San Mateo, CA, USA) with 41° field of view (FOV) lenses recorded video at 2.7 K resolution and 120 frames per second (fps) with a shutter speed of 1/1920s…”	________
(3b) Frame rate variability	If there was variable frame rate (e.g., Yes/No), the methods used to account for frame rate variability in analyses	PMID: 29175825, “All seven videos had an interlaced scan with frame rates of 25 and 30 Hz, making it possible to double the effective frame rates to 50 and 60 Hz. Videos were edited and deinterlaced using Adobe premiere Pro CS6 (Adobe Systems, San Jose, CA). The video display resolution was 1024 × 576 for cases 1 and 7; 704 × 480 for case 2; and 788 × 576 for cases 3–6.”	________
(3c) Shutter speed	The shutter speed of the camera used in recording	PMID: 34274559, “Eleven action cameras (HERO6; GoPro, Inc., San Mateo, CA, USA) with 41° field of view (FOV) lenses recorded video at 2.7 K resolution and 120 frames per second (fps) with a shutter speed of 1/1920s…”	________
(3d) Resolution	The image or video resolution (e.g., the number of pixels that make up the image by the horizontal and vertical axes)	PMID: 32095268, “…an additional camera with a 120° FOV GoPro Hero6 lens with 4 K resolution…” PMID: 33025319, “Resolution was assessed in terms of pixels per helmet…the resolution of the images ranged from 64 to 126,7000 pixels per helmet.”	________
(3e) Display or pixel aspect ratio	Ratio of width to height (in pixels) of an image	PMID: 32130020, “…16:9 aspect ratio…”	________
(3f) Data compression type	Method for image or video compression to reduce file size (e.g., Lossy or Loss-Less)		________
(3g) File format	The file format, which dictates compression algorithm (e.g., JPEG, MPEG-4); must report if fille formats were changed (i.e., file conversion)	PMID: 34274559, “Extract 3-s video clips (.mp4) and image sequences (.bmp) from a minimum of two camera views that captured the impact case. Extract a calibration image (.bmp) that corresponds with each of the aforementioned camera views.”	
4. Corrections Related to Video Quality			
(4a) Lens distortion correction	Methods/software used for correction for lens distortion	PMID: 30274537, “The effects of lens distortion were incorporated into the virtual view based on the lens profile of each camera.”	________
(4b) Motion blur	If motion blur occurs, the methods taken to make sure the point on the object being tracked is consistent between frames	10.1080/14763141.2018.1513059 “Edge definition was subjectively assessed based on qualities such as brightness, contrast, and presence of blur. Based on these factors, it was determined that the cameras at locations 2 and 4 provided the best footage for analysis.”	________
(4c) Unstable footage	The time of video removed due to unstable footage (e.g., unpredictable panning, zooming and movement); explain distortion management		
5. Data Processing			
(5a) Analysis software	Software used for analyses (e.g., ProAnalyst 3D, SynthEyes, PFTrack, Houdini, Nuke)	PMID: 32095268, “Three-second video clips from the two primary views were extracted and uploaded into head tracking software (PFTrack, The Pixel Farm, UK)…”	________
(5b) Data reduction/down sampling, if applicable	Initial and re-sampled frame rates as well as how it was managed		
(5c) Video stabilization	Methods/software used to correct for camera motion, such as panning and zooming	PMID: 30274537, “All camera views were ‘stabilised’ to remove the effects of camera movement, panning, tilting and zooming so that the background shown in each image remained stationary (Nuke X 10.0v1, Foundry, London, UK).”	
(5d) Filtering	Methods for filtering kinematic data obtained by video tracking	PMID: 33025319, “Position time histories were filtered using a 30 Hz, 4-pole Butterworth filter.”	
(5e) Start and end times	Method for determining the beginning and end of an impact	PMID: 33025319, “Start and End times of the impact were manually selected based on visual inspection of the slope of the translational velocity time histories alongside the game footage.”	
6. Data Reporting			
(6a) Accuracy	Validation procedures and accuracy of the measurement of interest	PMID: 32095268, “In a laboratory validation of our model-based image matching implementation, we determined the mean absolute errors in the estimated change in resultant translational velocity and rotational velocity (ΔVR and ΔωR, respectively) during simulated H2G and H2H impacts to be ± 0.24 m/s (± 10.7%) and ± 3.4 rad/s (± 21.8%), respectively (ref).”	________
(6b) Outcome measures	Primary outcome measures (e.g., object's orientation, velocity, acceleration, size, shape) and methods to calculate	PMID: 32095268, “Model-based image matching was used to reconstruct head translational velocity (*V*) and rotational velocity (*ω*) over time, similar to previously published methods (ref).” PMID: 33025319, “Velocities were calculated using the central difference method…”	________

**Table 4:** CHAMP 2022 checklist of information to include when reporting physical reconstruction studies of head acceleration events.

Checklist item	Explanation	Example(s)	Reported on Page No
1. Study Design and Case Selection			
(1a) Study purpose	The purpose of the study shall be explained clearly—e.g., Collect observational data, compare injury to controls, evaluate interventions	PMID: 21553135, “The objective of this study is to develop and introduce the concept of a new evaluation system that can be used to provide quantitative insight into the protective performance of football helmets against concussions.” PMID: 24844272, “The specific aims of the study were to (1) investigate the dynamics, impact location and kinematics, of no-injury and concussive impacts to the unprotected human head and (2) consider concussion tolerance values.”	________
(1b) Study design	The study design, e.g., case series, case–control, weighted sample, census		________
(1c) Case source material	The source of the actual events that are being reconstructed	PMID: 22012084**,** “A series of 10 events in which a catcher or umpire in Major League Baseball, who experienced a foul ball to the mask that resulted in a concussion, were analyzed through video and data on pitch characteristics.”	________
2. Surrogate Selection			
(2a) Surrogate used	The surrogate used: Whole ATD, headform, ATD components, use of neck	PMID: 16046355, “For the head to head tests (2–5 m/s), two biofidelic dummy headforms were used…We chose to use a Hybrid III automotive test dummy headform (Denton ATD Inc., Milan, OH)…The test was set up such that a Hybrid III head neck system (representing the struck player) was accelerated by gravity alone into contact with a stationary Hybrid III dummy (representing the striking player) that was suspended from an adjustable hoist assembly.”	________
(2b) Helmet/no helmet	Matching of the helmet model or equipment used in the tests to those used in the actual events being reconstructed	PMID: 29570748, “Samples of 37 football helmet models, including facemasks and chinstraps, were acquired as part of a series of impact performance tests. All models were either intended to be offered or still being used in the National Football League (NFL) for at least one season in the 2015–2017 time frame. Approximately 99% of current NFL players wear one of the helmet models included in this study. The helmet size that best fit the 50th percentile male Hybrid III dummy head was chosen for measurement.”	________
(2c) Headform type	The headform type, e.g., Hybrid III, NOCSAE, custom	PMID: 16046355, “We chose to use a Hybrid III automotive test dummy headform (Denton ATD Inc., Milan, OH) in the evaluation of the headgear because of its human-like response and the availability of literature correlating the head response to injury potential for a wide range of impact conditions. The head anthropometry approximates that of a 50th percentile adult male and has correct mass and mass moment inertial properties for proper dynamics.”	________
(2d) Biofidelity	Any modifications made to the headform to mount the wearable sensor, e.g., jaw, ear	PMID: 30802147, “a regular in-ear MV1 (MV1 in-ear) firmly placed in a tight canal on the HIII headform, representing an artificial ear canal…We created the canal by carving out a piece of the artificial skin covering the HIII headform. The tight canal’s diameter was slightly smaller than the sensor’s, only enough to allow the compliant properties of the rubber to expand and create a snug fit, mimicking real-life custom-molded placement.” PMID: 21994059, “The Hybrid III head was modified with an articulating mandible, dentition, and compliant temporomandibular joints (TMJ). It was instrumented for triaxial head acceleration and triaxial force at the TMJs and upper dentition. Mandible force and displacement were validated against cadaver impacts to the chin.”	________
(2e) Calibration of test surrogate	The biofidelity of the test components, especially custom components, including limitations in biofidelity, e.g., axial neck compress in HIII	PMID: 24920257**,** “For automotive crash testing, the Hybrid III head undergoes a calibration test wherein peak head acceleration must be between 210 and 260 g (a 50 g window) during a forehead impact following a drop of 10 in (25.4 cm) onto a flat steel plate (Federal Motor Vehicle Safety Standards FMVSS 49 CFR 572.6)….the repeatability for both headforms was within the implied repeatability of 5.3% derived from the Hybrid III’s forehead calibration test (assuming the 50 g window represents ± 2 SD; FMVSS 49 CFR 572.6).”	________
3. Test Apparatus			
(3a) General types	The type of test apparatus—e.g., Guided wire drops, monorail drop, rail-guided launcher, pneumatic impactor and belt-driven sleds	PMID: 29414471, “Shoulder impacts were reconstructed using a pneumatic linear impactor with an impacting arm of 13.1 kg with 67.79 mm of R338 vinyl nitile (VN) foam…Falls were reconstructed using a monorail drop rig with a modular elastomer programmer (MEP) anvil to simulate the playing surface.” PMID: 33000448, “Helmeted crash test dummies were launched into each other using two custom-made electric-powered belt-driven sleds (Fig. 1)…Each dummy was hung on a forward-facing steel pin that was connected to the sled carriage via an adjustable frame of T-slot extruded aluminum profiles. The pelvis was supported as well to maintain the position of the dummies as they were accelerated. The dummies were launched into each other by accelerating each carriage up to the desired pre-impact speed, then rapidly stopping it at the end of the sled track. As the carriage decelerated, the dummies slid off their sup- ports and struck each other helmet-first while airborne and under no external forces.”	________
(3b) Impact surface	The impact surface, both on the surrogate (e.g., Hybrid III ± helmet) and opponent, e.g., other surrogate, post, ice, playing surface, padded/unpadded. Effective stiffness should be described	PMID: 19440839, “During the resulting free fall drop, the ATD maintained its initial orientation to impact a padded load plate/anvil on its head….The anvil was fixed rigidly at the required location over a hardened concrete floor pit. The padding consisted of 2.54 cm (1 inch) thick Ensolite® foam (Gaska Tape (Australia) Pty Ltd).”	________
(3c) Closing speed	The closing speed(s) of impact and the basis for choosing closing speed	PMID: 29414471, “Ball-to-head impacts were performed by Clark and Hoshizaki (2016) using an air cannon which fired a lacrosse ball at 28.3 m/s (SD 2.2). This velocity was selected as a shot in women's lacrosse have been reported to have ball velocities up to 60mph (26.8 m/s) (Lincoln *et al*., 2007). Shoulder impacts were reconstructed using a pneumatic linear impactor with an impacting arm of 13.1 kg with 67.79 mm of R338 vinyl nitile (VN) foam (Rousseau and Hoshizaki, 2015). The inbound velocity was 5.0 m/s and chosen to reflect high-speed running of female soccer players (Krustrup *et al*., 2005; Mohr *et al*., 2008). Falls were reconstructed using a monorail drop rig with a modular elastomer programmer (MEP) anvil to simulate the playing surface. An inbound velocity of 4.5 m/s was selected by Clark and Hoshizaki (2016), after using Mathematical Dynamic Models (MADYMO) simulations to determine that a 1.57 m tall female being pushed forward at 1.0 m/s resulted in a head impact velocity of 4.5 m/s.”	________
(3d) Impact vector	Impact vector/location of test surrogate and impact partner and basis for choosing	PMID: 26117075, “Stereo high speed video of a single *in vivo* head impact was collected to estimate initial impact conditions (impact location on helmet and relative impact speed) that were used to set up a laboratory reconstruction of the impact…In the helmet and field frames, we computed the mean velocity of all 10 points on the helmet in the 10 frames (4 ms) before helmet contact. The difference between the helmet velocity vectors was used to determine the relative impact speed and orientation.” PMID: 22012084, “the impact location [of the baseball] on the mask was determined based on…four anatomic regions: forehead, eye- brow, nose, and chin. Each region is then further broken down into left, right, and center areas, except for region 10 (chin area). This analysis provided information about the general distribution of impacts over the mask of an umpire or catcher.”	________
(3e) Eccentricity	The velocity vector(s) for impacts relative to head. Are the impacts 'centric' (directed towards head center of mass), tangential or oblique (combination of centric and tangential)?	PMID: 22012084, “To best approximate the impact speed of a baseball from a foul tip, the plate speed of the baseball was examined…Initial pitch release speed and plate speed were recorded…A total of seven locations were investigated…referenced to the tip of the nose of the Hybrid III. The forehead location is targeted 9.2 cm (3 5/8 in.) above the tip of the nose…The three targets on the lateral edge of the face are 7.6 cm (3 in.) away from their corresponding mid-sagittal locations.”	________
(3f) Effective mass	Description of overall test fixture, surrogate and impact partner in the context of effective mass (e.g., headform rigidly attached to test frame, head and neck attached to sliding track). How was effective mass estimated?	PMID: 15670375, “Fig. 1 shows the reconstruction setup, which involved two Hybrid III male dummies. A helmeted head-neck assembly representing the struck player was attached to a 7.1 kg mass simulating the struck player’s torso and guided in free fall from a height to match the impact velocity determined from video analysis of the game collision…The [striking] mass was mStriking = 5.90 kg and included the Hybrid III head (3.64 kg), the load cell above the sensing element (0.34 kg), and the helmet with face mask (1.92 kg)….The mass of the struck player is mStruck = 8.40 kg and includes the head (4.38 kg), neck (1.06 kg), helmet and face mask (1.92 kg), and a portion of the torso mass (1.04 kg)…The effective mass of the striking player is (mEff.Striking = F/aStriking) On the basis of the average head acceleration and impact force, mEff.Striking = 14.0 kg, indicating a mass ratio of mEff.Striking/mStruck = 1.67, or a 67% greater effective mass of the striking player than that of the struck player during peak force.”	________
4. Instrumentation			
(4a) Linear head kinematics	The type (e.g., triaxial linear accelerometer) and model for instrumentation measuring linear kinematics	PMID: 30802147, “The HIII head was instrumented at its center of mass with an in-calibration triaxial linear accelerometer (Endevco; Meggitt Sensing Systems)…sampling at a rate of 20 kHz. Linear acceleration…data were filtered with a SAE CC1000 filter (DIAdem 2011)	________
(4b) Angular head kinematics	The type and model for instrumentation measuring angular kinematics (e.g., Clusters of linear accelerometers (3-3-3, 3-2-2-2), Angular rate sensors (angular velocity))	PMID: 16331164, “The Hybrid III was equipped with the standard triaxial accelerometers (Endevco 7264-2 k) at the head center of gravity (cg) and six more accelerometers in a 3-2-2-2 configuration to determine rotational acceleration.” PMID: 23697898, “a 50th percentile male Hybrid III head was instrumented with 3 Endevco 7264–2000 linear accelerometers (Endevco, Meggitt Sensing Systems, San Juan Capistrano, CA) to measure the head CM linear acceleration and 2 Kistler rotational accelerometers type 8838 and 8840 (TDAS, Diversified Technical Systems, Seal Beach, CA) to measure angular acceleration in the head’s sagittal (αy) and coronal (αx) planes, respectively.”	________
(4c) Range of instrumentation	The range of values able to be recorded by each piece of instrumentation. This shall be within the instrument’s measurement range	PMID: 21994059, “Three triaxial force sensors (Model 260A11, PCB Piezotronics, Depew, NY) were designed into the articulating mandible headform…The sensors had a sensing range of *x*–*y* = 2,220 N and *z* = 4,450 N.”	________
(4d) Sampling rate of instrumentation	The sample rate for each piece of instrumentation	PMID: 19440839, “Signals were conditioned and acquired at 20,000 Hz using a TDASPro (Diversified Technical Systems, Inc.) and later resampled at 10,000 Hz.” PMID: 23697898, “All data were acquired with a TDAS system at 20 kHz.”	________
(4e) Triggering	The trigger mechanism, e.g., contact switch, velocity gate, or head acceleration threshold, and how this functions with data acquisition	PMID: 15670375, “The following procedure was used to align time zero for the individual cases, because the orientations of the collisions and timing varied between tests. A “soft trigger” was used to determine the start of head acceleration. For most cases, a 1 g trigger was used to determine the start of the impact; however, some tests had noise on the responses requiring a 3 g…or 5 g trigger.” PMID: 23697898, “The drop velocity was measured using a double flag mounted on the drop assembly, passing through a photoelectric fork sensor, which also triggered data acquisition.”	________
(4f) Other instrumentation used	Type and model for instrumentation measuring quantities other than linear/angular kinematics (e.g., force, torque, pressure)	PMID: 21994059, “Three triaxial force sensors (Model 260A11, PCB Piezotronics, Depew, NY) were designed into the articulating mandible headform. One force sensor is situated between the upper dentition and the skull. The other two were placed at left and right TMJ.” PMID: 23697898, “The head was attached to a Hybrid III neck and instrumented with a 6-axes load cell at the upper neck. The free-falling head–neck assembly was synchronized to impact in the middle of the top of a horizontal moving aluminum striker plate. A Kistler 9257A triaxial piezoelectric force plate was sandwiched between the striker plate and the aluminum carriage.”	________
(4g) Calibration of instrumentation	Timing of last instrumentation calibration and/or the process of reference checks	PMID: 19440839, “A calibrated male 50th percentile Hybrid III ATD (Denton ATD, Inc.) was used….A full re-calibration of the dummy was performed after tests 5 and 15 [26 total tests completed].”	________
5. High-Speed Video			
(5a) Video recording parameters	The number and type of video recording devices (e.g., number of cameras, placement, resolution, and frame rate of video recordings)	PMID: 19440839, “Depending on the test series, one or two high-speed cameras (VisionResearch, Inc. – Phantom v4.0/v4.1/v4.3, 512 × 512 pixels at 1000 fps) recorded the impacts in the coronal and/or sagittal plane of the dummy.”	________
6. Data Processing			
(6a) Filtering	The filters used for processing each data stream and whether these comply with SAE J211, including any additional or alternative signal conditioning processes	PMID: 23697898, “All data were acquired with a TDAS system at 20 kHz and conditioned according to SAE J211 protocols, except angular acceleration (SAE 2007). Angular acceleration data were conditioned with an SAE Channel Class 180 filter.” PMID: 30802147, “Linear acceleration and angular velocity data were filtered with a SAE CC1000 filter and a SAE CC180 filter (DIAdem 2011; National Instruments), respectively, before computing a preliminary set of PLA and PRV values for each impact.”	________
(6b) Signal offset	The processes for identifying and removing signal offsets	PMID: 15739686, “Baseline offset in all signals was removed by subtracting the average of pre- trigger data samples from the entire signal.”	________
(6c) Calculated metrics used	The primary biomechanical outcome measures calculated (e.g., injury criteria)	PMID: 25533767, “Twelve existing kinematic injury criteria (Table 1) were calculated using the collected and processed 6DOF mouthguard measurement: Peak Translational Acceleration Magnitude…Head Injury Criterion (HIC15 and HIC36)…Severity Index (SI)…Peak Rotational Acceleration Magnitude…Rotational Injury Criterion (RIC)…Peak Change in Rotational Velocity Magnitude…Brain Injury Criterion (BrIC)…Head Impact Power (HIP)…Power Rotational Head Injury Criterion (PRHIC)…Generalized Acceleration Model for Brain Injury (GAMBIT)…Principal Component Score (PCS).”	________
(6d) Data transformation	The methods used to transform recorded data to analyzable data (e.g., numerical integration from angular velocity to angular acceleration, transformation from the location of the sensor to the center of gravity of the head)	PMID: 23604848, “ For physiological relevance, linear acceleration data are transformed from the mouthguard to the center of gravity of a 50th percentile male human head via the following equation: aCG = aMG + MG [x r] + ωMG x (ωMG x r), where aCG is the linear acceleration vector of the center of gravity of a 50th percentile male human head, aMG is the linear acceleration of the instrumented mouthguard, MG is the angular acceleration of the mouthguard, r is the vector from the accelerometer on the mouthguard to the center of gravity of a 50th percentile male human head, and ωMG is the angular velocity of the mouthguard. aMG is measured directly from the instrumented mouthguard accelerometer, and ωMG is measured directly from the instrumented mouthguard gyroscope. r is a constant vector which originates from the accelerometer on the instrumented mouthguard and projects posteriorly 105 mm, to the left 4 mm, and superiorly 54 mm. MG is computed by taking the derivative of ωMG using the five-point stencil method.”	________
(6e) Criteria for evaluating accuracy of reconstruction and best match	The method for determining which test best recreated the head impact and the process for evaluating success	PMID: 33000448, “The accuracy of the output of each reconstruction was assessed by comparing the translational and rotational velocity changes of both players’ helmets relative to the video analysis results.”	________
7. Test Matrix			
(7a) Repeatability assessment	Repeatability of the test apparatus across multiple tests and same input conditions. Repeatability may include inputs, e.g., velocities, and outputs, e.g., head accelerations	PMID: 24920257, “Repeatability was assessed using the COV, which equals the ratio of the standard deviation (SD) to the mean, expressed as a percentage…The COVs for PLA and PAA were calculated for each series of five repeated tests for all eight impact conditions in each lab.”	________
(7b) Sensitivity to input conditions	Process whereby the sensitivity of the outputs to the input conditions was assessed	PMID: 19440839, “The focus of the second aim was the ATD’s sensitivity to impact velocity and impact angle, on the upper and lower neck load measures and Nij…Upper neck moments were corrected according to Part 572E and the Nij was calculated for each test. The Nij(C7/T1) as proposed by Mertz *et al*.^19^ was calculated for the lower neck. Statistical treatment of the data was performed using the statistical software SPSS v15 (SPSS Inc.). Statistical significance for correlation between variables was assessed using Spearman’s test and measured against the level *α* < 0.01.”	________
8. Data Reporting			
(8a) Comparison of pre- and post-impact kinematics between event and reconstruction	Percent error in pre- and post- impact kinematics between the event being reconstructed and the reconstruction	PMID: 33000448, “The closing velocity between the dummy heads was − 5% ± 7% (average absolute error = 6%) of the target closing velocity determined from video analysis.”	________
(8b) Repeatability assessment	Results of repeatability assessment in terms of both input and output	PMID: 24920257, “Across both labs, the COVs for PLA indicated excellent repeatability (± 3%) for 13 and 14 of the 16 impact conditions for the MLSH and Hybrid III headforms respectively (Table 1). The remaining 5 impact conditions achieved acceptable repeatability (3% < COV < 7%). For peak angular acceleration (PAA), the repeatability for both headforms ranged from excellent to poor (Table 1).”	________
(8c) Sensitivity assessment	Influence of small variations in input on outcome parameters	PMID: 33000448, “Test results were also highly sensitive to small changes in impact location and path eccentricity, as would be expected in a collision between two spheroidal objects. This sensitivity was generally higher for players struck near the ‘‘equator’’ of the helmet as opposed to the top of the helmet. Particularly concerning was the observation in many cases of a tradeoff between input accuracy (Eq. 1) and output accuracy (Eq. 4).”	________

**Table 5:** CHAMP 2022 checklist of information to include when reporting computational studies of head acceleration events.

Checklist item	Explanation	Example(s)	Reported on Page No
1. Model Development			
(1a) Model selection	The model and version used in analyses; describe any modifications made to model parameters	PMID: 16284560, “The Wayne State University Head Injury Model (Version 2001) was used because…” PMID: 26192950, “On account of the presence of CSF between the meningeal membranes and the brain, a sliding-only contact definition was originally used for these interfaces. The contact definition was, however, found to be incompatible with any currently available MPP versions of LS-DYNA, and since the computational time on a single computational node for the complete THUMS-KTH model together with the vehicle model was considered too long, a tied interface was used instead.”	________
(1b) Model reference	The geometry of the model	PMID: 24735430, “The DHIM was created based on a template high-resolution T1-weighted MRI of a person selected from the group of concussed athletes whose head was positioned neutrally without tilting in the MRI.” PMID: 2343473, “The geometry of the model was determined by computer tomography, magnetic resonance imaging, and sliced color photos, which were available through the Visible Human Database.”	________
(1c) Brain structures	The structures included in the brain model	PMID: 26762217, “…initial model development combined the label maps to include only four distinct parts: cerebrum (combined white and gray matter), cerebellum, CSF and ventricles.”	________
(1d) Model elements	Elements used for meshing the brain (e.g., hexahedral meshes)	PMID: 24065136, “…hexahedral brain meshes were developed with feature-based blocking technique using ANSYS ICEM CFD/HEXA 12.0.”	________
(1e) Number and size of elements in model	The number of elements used to define the brain model	PMID: 24735430, “In total, the model contains 101,420 nodes and 115,228 elements with a combined mass of 4.562 kg for the head, and 56,632 nodes and 55,062 elements with a combined mass of 1.579 kg for the brain (1.558 kg without the spinal cord). The average element size for the whole head and the brain is 3.2 ± 0.94 mm and 3.3 ± 0.79 mm, respectively.”	
(1f) Solver	The method used for time integration (e.g., LS-DYNA or ABAQUS solvers)	PMID: 24529781, “The FE solver used in this study was the explicit LS-DYNA_971_7600 code.”	________
(1g) Brain material properties	Assignment of brain and membrane structures material properties (e.g., linear viscoelastic, nonlinear, hyper-viscoelastic) and values (e.g., Young's modulus, density and Poisson's ratio for bone; constants for viscoelastic constitutive laws)	PMID: 24063789, “Visco-elasticity was assumed for brain material model and the skull was modeled by a three layered composite shell representing the inner table, the dipole and the external table of human cranial bone.”	________
(1h) Skull-brain interface	Boundary conditions between brain and skull and among internal structures (e.g., tied and/or connected or nodal sharing)	PMID: 17096222, “The interface between the skull and the brain was modeled in three different ways ranging from purely tied (no-slip) to sliding (free-slip).”	________
2. Model Validation			
(2a) Validation reference	Medium from which experimental data were collected (e.g., human subjects, cadavers)	PMID: 32240424, “Head rotation in these experiments was induced by directly striking or stopping a cadaveric head…”	________
(2b) Validation method	Methods used for collecting experimental data used to validate the brain’s responses (e.g., *in vivo*, cadaveric, using neutral density targets, using marker-based strains)	PMID: 28701050, “In the cadaver impact experiments, local displacements were evaluated throughout the brain using a high-speed biplanar X-ray system to track the relative motion of implanted radio-opaque neutral density targets.”	
(2c) Impact direction	Direction of the applied loading conditions for model validation experimental data	PMID: 28394205, “We simulated the scenario that resulted primarily in rotation about the axial plane…”	________
(2d) Impact magnitude	Magnitude of the applied loading conditions for model validation experimental data	PMID: 22992118, “…the impactor mass was 5.59 kg and the impactor velocity was 9.94 m/s.”	________
(2e) Impact duration	Duration of the applied loading conditions for model validation experimental data	PMID: 34863650, “A typical impact of 100 ms…”	________
(2f) Validation analyses	Methods used for comparing model data to experimental data	PMID: 30608998, “CORrelation and Analysis (CORA) and Normalized Integral Square Error (NISE) are employed to evaluate model validation performance for both brain strain and brain-skull relative motion.”	________
3. Model Simulation			
(3a) Model dimensions	Scaling of the model dimensions to match subject or why a representative set of dimensions is appropriate	PMID: 32240424, “For each cadaveric impact, the WHIM was scaled along the three anatomical directions to match with the reported head dimensions”	________
(3b) Simulation data	Description of the simulation data	PMID: 33126836, “The samples included 110 head impacts measured in a variety of contact sports at Stanford University (ref) and their two batches of augmented data sets (*n* = 1320, 110 × 6 × 2), 53 head impacts reconstructed from the NFL (ref) and their four batches of augmented data sets (*n* = 1272, 53 × 6 × 4), and 314 impacts recorded in American high-school football (ref).” PMID: 18278592, “Detailed descriptions of the game film selection and analysis can be found in Pellman *et al*. (2003a, 2003b), while the details regarding the accident reconstruction methodology can be found in Newman *et al*. (1999, 2000, 2005).”	________
(3c) Simulation efficiency	Methods used to enhance simulation efficiency	PMID: 31758002, “In this study, we developed a deep convolutional neural network (CNN) to train and instantly estimate impact-induced regional brain strains with sufficient accuracy.”	
(3d) Simulation runtime and hardware platform	Wall clock runtime and computing hardware platform	PMID: 24735430, “All impact simulations were executed on a Linux computer (Intel Xeon E5-2698 with 256 GB memory). A typical impact of 100 ms required ~ 6 h for simulation with Abaqus/Explicit (double precision).”	
(3e) Strain sensitivity to impact kinematics	Methods used to determine how impact kinematics affect simulation outcomes	PMID: 24610384, “Because the focus of our study is to examine the sensitivity of strain-related responses to alin and arot, the *θ* and *α* angles characterizing the translational and rotational axes were clustered. A linear regression for each regional output variable (three variables in four ROIs) was performed based on the 100 impact simulation results from each head FE model. An additional linear regression was performed using νrot as the single independent variable, and their performances were compared in terms of coefficients of determination (R2). Finally, Pearson correlation was performed between the two FE models to assess the similarity in their responses relative to head impacts.”	________
4. Data Reporting			
(4a) Outcome measures	Metrics used to evaluate model simulation data (e.g., 95th percentile maximum principal strain), including their calculation (e.g., strain rate)	PMID: 24077860, “The five brain mechanical variables used for comparisons were the maximum principal strain (*ε*), maximum principal strain rate (*ε*.), their product (*ε* × *ε*.), von Mises stress (*δ*), and pressure (*P*).” 10.1016/j.brain.2021.100024, “the axonal strain rate is the logarithmic strain rate component resolved in the direction of fiber alignment”	

We suggest that authors, peer-reviewers, and journal editors refer to these checklists as the “CHAMP 2022 Reporting Guidelines” and use them in describing studies of head acceleration measurement in the peer-reviewed literature. Manuscript structure should follow specific journal instructions and stylistic requirements for authors. Authors should simply report checklist items within the article with enough detail for reviewers, authors and readers to discern study rigor. We suggest authors who wish to cite CHAMP 2022 checklists should cite this manuscript. If a journal supports CHAMP 2022, it can cite these manuscripts in their “Instructions for Authors” and require submission of the relevant checklist along with identification of the page number on which each item is reported.

The CHAMP 2022 guidelines will likely evolve and are not all-encompassing. We encourage professional organizations to collaborate on updates to checklist items that warrant revision as the science and technology of head kinematic measurement continues to develop.

## Industry Involvement in Research

In addition to the checklists presented above, another important aspect of Transparency and Disclosure in head acceleration measurement studies is the involvement and/or relationship of the study authors to head kinematic sensor manufacturers or device suppliers. Most scientific journals require authors to disclose real and perceived conflicts of interest, as well as sources of funding related to the research, to allow the reader to evaluate real or potential bias. However, in head acceleration measurement studies, a company’s involvement may extend beyond the provision of in-kind or financial support for a study, and its role may be more nuanced than in other fields (See Table 6 for examples). It is important to note that disclosing a company’s involvement in study funding, design, analysis, or interpretation of data does not necessarily mean the study is biased; instead, disclosure is key to transparency. Specifically, disclosure may promote trust by assuring the readers there are no hidden conflicts of interest influencing the research. Therefore, disclosure of all sources of support, including the six types listed in Table 6 is a critical component of head acceleration measurement studies and should be considered a key aspect of CHAMP 2022 reporting guidelines.

**Table 6:** Potential sources of bias resulting from research partnership.

(1) funding for sensor validation/testing,
(2) funding for sensor implementation,
(3) in-kind equipment for study use,
(4) aid in study design and development,
(5) proprietary software for data cleaning and analysis,
(6) help in data analysis, interpretation and dissemination

## Summary

The Consensus Head Acceleration Measurement Practices (CHAMP) group was founded to develop and recommend best practices for the collecting, analyzing, and reporting of head acceleration measurement data in sport. Comprised of a diverse group of scientists, the CHAMP group, through its workgroups, developed consensus methodologies and companion summary statements which were discussed, revised and voted upon at the CHAMP conference in March 2022 and are summarized in the companion manuscripts in this series. Herein, we summarize the motivation and methods of the consensus process and introduce recommended reporting checklists to be used to increase transparency and rigor of future experimental design and publication of work in this field. The checklists provide an accessible means by which to: (a) translate the rich details of best practice summarized in the other manuscripts in this series; (b) improve the reporting of studies utilizing head acceleration measurement in sport and military applications; and (c) evaluate and interpret published work in this field. Aligned with the goal of improving the rigor, quality and consistency of research in this area, they also serve as a tool for authors as they prospectively consider design of their study.

## Supplementary Information

Below is the link to the electronic supplementary material.

Supplementary file1 (PDF 543 kb)
