# Supplementary material for: Consensus Head Acceleration Measurement Practices (CHAMP): Origins, Methods, Transparency and Disclosure
Source: Ann Biomed Eng. 2022 Aug 3;50(11):1317–45. doi: 10.1007/s10439-022-03025-9 (PMC9652170; doi:10.1007/s10439-022-03025-9)
Supplement: Supplementary file 1 — Supplementary file1 (PDF 543 kb) [file 10439_2022_3025_MOESM1_ESM.pdf]

## Appendix 1: Participants

**CHAMP leadership group:**

- Kristy Arbogast, PhD – Children's Hospital of Philadelphia (CHOP) and University of Pennsylvania
- Jeff Crandall, PhD – Biocore, LLC
- James Funk, PhD – Biocore LLC
- Gary Solomon, PhD – National Football League Player Health and Safety

**Workgroup leaders:**

- Transparency and Disclosure
    - Kristy Arbogast, PhD – CHOP/University of Pennsylvania
    - Jaclyn Caccese, PhD – Ohio State University
- Study Design and Statistical Analysis in Studies of Head Acceleration Measurement
    - Steve Rowson, PhD – Virginia Tech
- Laboratory Validation of Wearable Head Kinematic Devices
    - Lyndia Wu, PhD – University of British Columbia
    - Lee Gabler, PhD - Biocore, LLC
- On-Field Validation and Use of Wearable Head Kinematic Devices
    - Jillian Urban, PhD – Wake Forest University
    - Calvin Kuo, PhD - University of British Columbia
    - Initial contributions by Nate Dau, PhD – formerly of Biocore, LLC
- Video Analysis of Head Acceleration Events

    - William Neale, MArch – J.S. Held
- Physical Reconstruction of Head Acceleration Events
    - James Funk, PhD – Biocore, LLC
- Computational Modeling of Head Acceleration Events
    - Joel Stitzel, PhD – Wake Forest University
    - Songbai Ji, PhD – Worcester Polytechnic Institute

**Attendees – March 2022 CHAMP conference (in-person and virtual):**

| Last name | First name | Affiliation |
|---|---|---|
| Arbogast | Kristy | Children’s Hospital of Philadelphia/University of Pennsylvania |
| Arrington | Dusty | Adamson Engineering, LLC |
| Bartsch | Adam | Prevent Biometrics |
| Begonia | Mark | Virginia Tech |
| Bergmann | Jeroen | University of Oxford |
| Broglio | Steven | University of Michigan |
| Buckley | Thomas | University of Delaware |
| Bussey | Melanie | University of Otago |
| Caccese | Jaclyn | Ohio State University |
| Chu | Jeff | Simbex |
| Crandall | Jeff | Biocore LLC |
| Esquivel | Amanda | University of Michigan - Dearborn |

| | | |
|---|---|---|
| Falvey | Eanna | World Rugby |
| Funk | James | Biocore LLC |
| Gabler | Lee | Biocore LLC |
| Ghajari | Mazdak | Imperial College London |
| Goodger | Drew | Prevent Biometrics |
| Greenwald | Richard | Simbex |
| Harezlak | Jaroslaw | Indiana University |
| Harmon | Kimberly | University of Washington |
| Hawes | Damien | HITIQ |
| Heiderscheit | Bryan | University of Wisconsin |
| Huber | Colin | University of Pennsylvania |
| Ide | Thad | Riddell Sports |
| Ji | Songbai | Worcester Polytechnic Institute |
| Jones | Ben | Leeds Beckett University |
| Kaminski | Thomas | University of Delaware |
| Kelshaw | Patricia | University of New Hampshire |
| Kleiven | Svein | KTH Royal Institute of Technology |
| Kraft | Reuben | Penn State University |
| Kuo | Calvin | University of British Columbia |
| Laudenbach | Tom | HITIQ |
| Luck | Jason | Duke University |

| | | |
|---|---|---|
| Lynall | Rob | University of Georgia |
| Makdissi | Michael | Australian Football League |
| Mao | Haojie | Western University |
| Mayer* | Thom | NFL Players Association |
| McIntosh | Andrew | McIntosh Consultancy & Research |
| Moran | Ryan | University of Alabama |
| Neale | William | JS Held |
| Panzer | Matthew | University of Virginia |
| Patton | Declan | Children's Hospital of Philadelphia |
| Reynier | Kristen | University of Virginia |
| Rooks | Tyler | U.S. Army Aeromedical Research Laboratory |
| Rowson | Steve | Virginia Tech |
| Schmidt | Julianne | University of Georgia |
| Shogren | Mike | Prevent Biometrics |
| Siegmund | Gunter | MEA Forensic Engineers & Scientists |
| Sills* | Allen | NFL |
| Smith | Terry | Galeatus LLC |
| Stitzel | Joel | Wake Forest University |
| Tierney | Gregory | Ulster University |
| Urban | Jillian | Wake Forest University |
| Willinger | Remy | University of Strasbourg |

| | | |
|---|---|---|
| Willmott | Catherine | Australian Football League |
| Withnall | Chris | Biokinetics and Associates Ltd. |
| Wu | Lyndia | University of British Columbia |

*observers only

**Appendix 2: Voting results for each summary statement**

| Summary statements | Number voting | Voting results |
|---|---|---|
| **1. Laboratory Validation of Wearable Head Kinematic Devices** | | |
| 1.1. A wearable device that measures head kinematics must be independently validated for its intended application through controlled laboratory testing, and the laboratory should simulate the real-world loading environment in which the device will be used.* | 33 | 100% support |
| 1.2. Laboratory testing of wearable devices should use a validated biofidelic anthropomorphic test device (ATD) headform combined with a repeatable and reproducible test setup that enables testing across multiple levels of magnitude, duration and direction that simulate on-field linear and angular head kinematics relevant to the setting of study.* | 35 | 97% support |
| 1.3. Reference sensor setup and validation metric selection depend on the intended application of the wearable device, which can vary on four main levels: impact counting, impact magnitude, impact direction, and the | 33 | 97% support |

| | | |
|---|---|---|
| time-history measurement of six-degree-of-freedom (6DOF) head kinematics. | | |
| 1.4. If a wearable device is designed to measure and report metrics derived from head kinematics, ground truth measurements must be collected with an ATD-embedded laboratory-grade reference sensor system. If a wearable device is designed to count impacts only, a reduced reference setup enabling verification of impact events may be applied. | 35 | 86% support |
| 1.5. Processed data from the wearable device must be compared with ground truth measurements using validation metrics and statistical methods that enable complete, unbiased, and application-relevant assessment of accuracy and uncertainty. | 35 | 83% support |
| **2. On-Field Validation and Use of Wearable Head Kinematic Devices** | | |
| 2.1. A head acceleration event (HAE) is defined as an event/incident that gives rise to an acceleration response of the head caused by an external short-duration collision force applied directly to the head or indirectly via the body in sport, recreational, military, or other activities of interest. Wearable devices are often both kinematically | 32 | 91% support |

| | | |
|---|---|---|
| and field validated for direct HAEs and not indirect HAEs due to the limitation of reproducing indirect HAEs in the lab and identifying indirect HAEs on the field, respectively. | | |
| 2.2. Kinematic data must be filtered to remove potential false positive recordings and verify valid HAEs. Data windowing, video verification, and pre- and post-processing techniques aid in data validation. Individual verification of HAEs is challenging, and time consuming but improper data validation may lead to errors in estimation of exposure.* | 31 | 100% support |
| 2.3. Video review serves as an independent verification of HAEs for a given application (e.g. device development, sport setting) and provides contextual information for HAEs. However, video should not be considered ground truth as the confidence in video review depends on video quality and a robust labelling process. Guided and blinded video review of head acceleration events are useful components to device performance in an on-field environment.* | 32 | 100% support |
| 2.4. Advanced processing techniques (e.g. algorithms or hardware solutions) have the potential to offer fast and reliable verification of valid HAEs. However, they are often | 32 | 100% support |

| | | |
|---|---|---|
| developed for specific wearable devices in specific applications (e.g., collegiate football) and it is best practice to independently validate processing methods for use in the intended application.* | | |
| 2.5. Before deploying head acceleration measurement devices in an on-field environment, users should establish data collection and analysis protocols according to the activity, resources, and research questions. Additionally, users should ensure 1) the devices are functional, 2) the batteries are charged, 3) the devices are attached securely to the individual, and 4) the wearable device is time-synchronized with other concurrent data sources (e.g. video, GPS systems).* | 33 | 100% support |
| **3. Physical Reconstruction of Head Acceleration Events** | | |
| 3.1. Physical reconstructions are a valuable methodology for understanding the dynamics of head acceleration events captured on video. | 32 | 97% support |
| 3.2. Due to selection bias and a small sample size, physical reconstruction study designs are typically limited to case series and case-control studies. | 31 | 100% support |

| | | |
|---|---|---|
| 3.3. Common test surrogates (Hybrid III dummy, NOCSAE headform) are limited in their ability to match the size, stiffness, posture, and active muscle tension of a particular athlete in a reconstruction. | 32 | 88% support |
| 3.4. The test apparatus in a physical reconstruction should be designed to recreate the 3-D interaction of the head and collision partner by matching their effective masses, combined stiffness (including head protection), closing speed, path eccentricity, and impact locations. | 31 | 90% support |
| 3.5. When conducting physical reconstructions, iterative testing should be performed to match video-derived parameters, both pre- and post-impact, and assess the repeatability and sensitivity of the test results to variance in test conditions.* | 30 | 100% support |
| 3.6. Researchers should identify and quantify the biomechanical parameters of interest and evaluate the accuracy, repeatability, and sensitivity of the reconstruction process relative to the effect size of the biomechanical parameters.* | 29 | 100% support |
| **4. Video Analysis of Head Acceleration Events** | | |

| | | |
|---|---|---|
| 4.1. Video analysis and videogrammetry are critical tools to understand the position, orientation, and motion of objects observable in video, such as the head or body of a sports athlete, or the helmet and equipment positioning and orientation. Analysis of these positions and orientations can be used to calculate change in linear and rotational velocities of the head as a result of an impact event. These techniques and procedures expand beyond sports and are applicable to the measuring of motion and position of most anything observable in video. | 24 | 92% support |
| 4.2. When applied properly, and validated, current video tracking methods have been shown to accurately estimate pre and post impact velocities. These estimates can be used to calculate characteristics of impact severity, such as change in velocity, using industry standard methodologies. | 24 | 83% support |
| 4.3. There are several variables (e.g. frame rate, resolution) that can affect the quality of the results of video analysis. The effect these variables have on the quality of the analysis should be quantified when possible, establishing a range of certainty for the specific set of parameters used in a given analysis.* | 26 | 100% support |

| | | |
|---|---|---|
| 4.4. Videogrammetry provides valuable input when reconstructing head acceleration events in the laboratory or when performing multi-body or finite element modeling.* | 29 | 100% support |
| 4.5. Videogrammetry should consider and correct, when possible, sources of error including lens distortion, interlacing, down sampling, compression, and variance in timestep.* | 25 | 100% support |
| **5. Computational Modeling of Head Acceleration Events** | | |
| 5.1. Brain biomechanical models have strong potential to improve injury prediction and interpret impact exposure over impact kinematics alone. They provide the ability to interrogate physics-based tissue level response, estimate risk of injury, and offer insight into injury specifics such as location and extent of structural damage.* | 32 | 100% support |
| 5.2. The modeling community advocates for the sensor community to standardize reporting of head kinematic data. Standardized reporting should include sensor hardware and software details as well as specifics on coordinate system, post-processing, sampling frequency, and subjects' morphological and demographic information.* | 33 | 100% support |

| | | |
|---|---|---|
| 5.3. It is recommended model quality be assessed comprehensively by comparing with experimental data related to the metric used for model predictions (e.g. deformation, strain, stress), correlating against real-world data, and then where possible, comparing to responses from existing models. It is also recommended that models be reevaluated for validation quality when new experimental data or analytic strategies become available.* | 32 | 97% support |
| 5.4. We recommend modelers explore modern data science techniques to efficiently process large amounts of sensor impact data. | 32 | 94% support |
| 5.5. The modeling community advocates for a curated open-access database repository to facilitate sharing of real-world data such as subject-specific head kinematics, injury diagnoses, and other associated information including head/brain morphology. In addition, simulation results from existing models using idealized kinematic profiles should be shared as a benchmark for cross-model examination.* | 32 | 94% support |
| **6. Study Design and Statistical Analyses** | | |

| | | |
|---|---|---|
| 6.1. Head impact sensor studies are typically observational in design, which limits their conclusiveness because they are easily contaminated by unseen confounding factors and biases. Investigators should be wary of selection and sampling biases when composing their samples. Efforts should be made to measure and account for suspected confounders. | 36 | 83% support |
| 6.2. Head impact sensor studies benefit from multidisciplinary teams with essential expertise. In addition, establishing partnerships with the research participant community can help produce more representative and reliable conclusions. | 36 | 89% support |
| 6.3. Proper operational planning for sensor maintenance and technical failures will help minimize missing data. In addition, video recording of data collection sessions is recommended as a resource for explaining and verifying impact events as needed. | 36 | 86% support |
| 6.4. Data quality must be assessed for outliers and spurious data and addressed through data cleaning practices. Suspected missing data should be noted, and all kinematic waveforms should be inspected, either computationally or | 34 | 91% support |

| | | |
|---|---|---|
| manually. Sensor validity should dictate the necessity and scope of these practices.* | | |
| 6.5. Investigators should employ analysis techniques that minimize sampling bias effects. Further, we recommend statistical transparency in both procedure and output.* | 33 | 94% support |
| 6.6. Investigators should perform a common-sense check on the data and their analysis results. Care should be taken to investigate results that appear to be inconsistent, unrealistic or counterintuitive. Explanations and disclosures of disparities with reality will help inform better data collection, cleaning, and analysis techniques.* | 29 | 93% support |

*Revised from initial draft after group discussion

**Appendix 3: Disclosures – March 2022 CHAMP conference participants and work group members**

| | | |
|---|---|---|
| **Support for attendance or participation in the conference content or activities** | K. Arbogast | Football Research Inc., payments made to institution |
| | A. Bartsch | Prevent Biometrics |
| | M. Bussey | World Rugby, University of Otago |
| | J. Caccese | NFL, travel funds for attendance |
| | J. Crandall | NFL and Football Research Inc., payments made to Biocore |
| | J. Funk | NFL and Football Research Inc., payments made to Biocore |
| | L. Gabler | NFL and Football Research Inc., payments made to Biocore |
| | K. Harmon | Gift fund to institution support concussion research |
| | D. Hawes | HITIQ |
| | T. Ide | Riddell |
| | S. Ji | NIH, NSF |
| | R. Kraft | NSF |
| | T. Laudenbach | HITIQ |
| | T. Smith | Galeatus LLC |

| | | |
|---|---|---|
| **Grants or contracts from entities (government support excluded)** | K. Arbogast | Football Research Inc., Chuck Noll Brain Research Foundation (payments to institution) |
| | A. Bartsch | World Rugby |
| | J. Bergmann | Lab10x; Podium Analytics Youth Sport Centre at University of Oxford |
| | S. Broglio | NCAA (payments to institution) |
| | T. Buckley | State Space Labs, Henry M. Jackson Foundation for the Advancement of Military Medicine, NCAA/DoD CARE Grand Alliance (payments to institution); University of Nevada COBRE (payment to individual). |
| | M. Bussey | World Rugby |
| | J. Caccese | American College of Sports Medicine (payment to institution). |
| | J. Chu | Riddell |
| | J. Crandall | Football Research, Inc. (contract to Biocore), NFL (contract to Biocore) |
| | R. Greenwald | Riddell |
| | J. Harezlak | NCAA |
| | K. Harmon | Football Research Inc. (payment to institution) |
| | D. Hawes | HITIQ |

| | | |
|---|---|---|
| | B. Heiderscheit | Football Research Inc., NFL, GE Healthcare, Rheon (payments to institution) |
| | P. Kelshaw | USA Lacrosse, NOCSAE |
| | T. Laudenbach | HITIQ |
| | R. Lynall | NOSCAE, Andee's Army, Georgia Clinical and Translational Science Alliance, NFL and Football Research Inc. (payments to institution) |
| | J. Mihalik | NCAA, NFL, Football Res. Inc. (payments to institution) |
| | R. Moran | Southeast Athletic Trainers Association, Football Research Inc. (payments to institution). |
| | M. Panzer | Diversified Technical Systems, Inc. |
| | D. Patton | Football Research Inc. (payment to institution) |
| | S. Rowson | NCAA, NOCSAE (payments to institution). |
| | J. Schmidt | NCAA (payments to institution) |
| | M. Shogren | World Rugby |
| | J. Stitzel | Childress Institute for Pediatric Trauma, NASCAR, Toyota Racing Development (payments made to institution) |
| | G. Tierney | World Rugby and Rugby Football League |

| | | |
|---|---|---|
| | J. Urban | Childress Institute for Pediatric Trauma, Toyota Racing Development (payments made to institution) |
| | C. Withnall | Football Research Inc. |
| | R. Willinger | Humanetics |
| **Royalties or licenses** | A. Bartsch | Cleveland Clinic, Prevent Biometrics |
| | J. Chu | For Riddell Insite and Sideline Response System |
| | R. Greenwald | For Riddell Insite and Sideline Response System |
| | M. Shogren | Cleveland Clinic |
| | R. Willinger | Humanetics |
| **Consulting fees** | K. Arbogast | NFL Players Association, grant reviews |
| | D. Arrington | Various defense firms, reconstruction of vehicle and pedestrian collisions |
| | S. Broglio | Medicolegal, grant reviews |
| | T. Buckley | Grant Reviews |
| | J. Crandall | NASCAR Head Neck and Spine Committee |
| | J. Funk | Football Research Inc. |
| | B. Heiderscheit | Altec, Inc, Biocore LLC |
| | R. Lynall | Biocore LLC via UNC Chapel Hill |

| | | |
|---|---|---|
| | A. McIntosh | Self-employed consultant. Consulting fees from insurance companies, law firms, Racing Australia, Transport for New South Wales, AFL, Worksafe Victoria, IOC, and Cricket Australia. |
| | G. Solomon | NFL |
| **Payment or honoraria (lectures, presentations, speaker bureaus, manuscript writing, educational events)** | K. Arbogast | American Academy of Pediatrics |
| | T. Buckley | Journal of Sport and Health Sciences – Editorial Board, Precision Athletic Training, Eastern Athletic Trainers Association, Pennsylvania Athletic Trainers Society, Shandong Sport University |
| | J. Caccese | Child Neurology Society, Eastern Athletic Trainers Association. |
| | J. Funk | Football Research Inc. |
| | K. Harmon | NFL Head, Neck and Spine Committee |
| | R. Moran | Southeast Athletic Trainers Association, National Athletic Trainers Association, Mississippi Athletic Trainers Association (MATA) |
| | S. Rowson | NOCSAE Scientific Advisory Committee |
| | J. Urban | International Association for Dance Medicine & Science |
| | T. Smith | Hockey Equipment Certification Council |

| | | |
|---|---|---|
| **Payment for expert testimony** | D. Arrington | Various defense firms, reconstruction of vehicle and pedestrian collisions |
| | J. Funk | Expert witness work, sometimes related to head injury. |
| | A. McIntosh | Expert testimony on the causation of head injuries in criminal and civil legal matters. Fees paid to company. Very few, if any of these matters, involve concussion in sport. |
| | G. Siegmund | MEA Forensic Engineers & Scientists (am a salaried employee and owner of the firm). Fees paid to company. None of my forensic cases involve wearable sensors. |
| **Support for attending meetings or travel (government support excluded)** | K. Arbogast | NFL Players Association, Football Research Inc., NOCSAE Scientific Advisory Committee |
| | A. Bartsch | Prevent Biometrics, Department of Defense |
| | M. Bussey | University of Otago |
| | J. Caccese | American College of Sports Medicine, Child Neurology Society, Eastern Athletic Trainers Association. |
| | J. Crandall | Football Research Inc., NFL, Biocore |
| | J. Funk | NFL |
| | L. Gabler | Football Research Inc., NFL, Biocore |

| | | |
|---|---|---|
| | K. Harmon | NFL Head, Neck, and Spine Committee |
| | B. Heiderscheit | NFL Soft Tissue Injury Task Force |
| | R. Lynall | NATA, University of Georgia |
| | A. McIntosh | IOC |
| | M. Shogren | Prevent Biometrics |
| | G. Siegmund | MEA Forensic Engineers & Scientists |
| | C. Willmott | Australian Football League, Monash University |
| **Patents, issued and pending** | A. Bartsch | 2011278996, 2011278997, 2011278999, 2012219306, 2019379578, 2019404197, 21183467.6, 19817851.9, 19845637.8, 2,805,252, 2,805,250, 2805266, 2,837,239, EP2593010, 3338631, 2593015, 2592998, EP2675356, EP2593010, 2593015, 2592998, 16/781,119, 16/737,325, 16/682,767, 16/720,589, 15/432,107, 63/170,217, 63/181,574, EP2675356, EP2593010, 2593015, 602011054987.1, EP2675356, EP2593010, 2593015, 502019000006365, EP2593010, 2593015, 2592998, EP2593010, 2593015, 2592998, EP2675356, 9,149,227, 10,582,883, 9,289,176, 9,044,198, 9,585,619 (Prevent Biometrics, Cleveland Clinic). |

| | | |
|---|---|---|
| | J. Bergmann | WO 2019/020969/ US20200155033/ EP3658009; WO 2021/111132 |
| | S. Broglio | U.S. Application No. 17/164,490 - pending |
| | J. Chu | All IP related to InSite and Sideline Response System assigned to Riddell |
| | J. Crandall | U.S. Patent 20190110746 - U.S. Patent 2019010584 - Co-inventor, Biocore ownership |
| | R. Greenwald | All IP related to InSite and Sideline Response System assigned to Riddell |
| | D. Hawes | Australian Innovation Patent (Patent No. 2021107530 and Patent No. 2021107528) |
| | T. Ide | Riddell (Riddell owns Head Acceleration Measurement patents). |
| | T. Laudenbach | Australian Innovation Patent (Patent No. 2021107530 and Patent No. 2021107528) |
| | J. Luck | US 2016/0345903 |
| | M. Shogren | US Patent 9,149,227, US Patent 9,585,619, US Patent 10,582,883, AU Patent 2011278996, AU Patent 2011278997, AU Patent 2011278999, AU Patent 2012219306, CA Patent 2,805,252, CA Patent 2,805,250, CA Patent 2,837,239, CA Patent 2,805,266, |

| | | |
|---|---|---|
| | | CA Patent 2,907,745, EP Patent 2,593,010, EP Patent 2,675,356, EP Patent 3,338,631, EP Patent 2,593,015, EP Patent 2,592,998. Other patents pending |
| | G. Siegmund | US Patent No. 10,548,510 |
| | J. Stitzel | 2 patents pending on mouthpiece-based head acceleration measurement technology |
| | J. Urban | Patent pending for mouthpiece-based head acceleration measurement technology |
| | L. Wu | U.S. Patent 10,172,555. US PCT Patent Application PCT/US2019/066088 – Inventor, Patent applications assigned to Stanford University. |
| **Participating in DSM Board or Advisory Board** | K. Arbogast | NOCSAE Scientific Advisory Committee |
| | J. Crandall | NASCAR Head, Neck and Spine Committee |
| | S. Rowson | NOCSAE Scientific Advisory Committee |
| **Leadership or fiduciary role in relevant entity, other board, society, committee or advocacy grp. Paid or unpaid** | K. Arbogast | NFL Engineering Committee and NFL Sensor Committee |
| | J. Bergmann | Lab10X |
| | M. Bussey | Sport and Exercise Science New Zealand |
| | J. Crandall | NFL Engineering Committee |
| | K. Harmon | NFL Head, Neck and Spine Committee, Pac-12 Brain Trauma Task Force |

| | | |
|---|---|---|
| | D. Hawes | HITIQ |
| | B. Heiderscheit | NFL Soft Tissue Injury Task Force |
| | T. Laudenbach | HITIQ |
| | J. Luck | The Ohio State University Injury Biomechanics Symposium |
| | A. McIntosh | Standards Australia committees ISO working group on occupational protective helmets. |
| | J. Mihalik | Senaptec, Inc - Co-Founder; Chief Science Officer |
| | M. Shogren | Prevent Biometrics |
| | J. Stitzel | Elemance, LLC |
| | C. Willmott | Australian Football League, Monash-Epworth Rehabilitation Research Centre Advisory Board |
| **Stock and stock options** | A. Bartsch | Prevent Biometrics |
| | J. Bergmann | RegMetrics |
| | J. Crandall | Biocore LLC – Owner |
| | D. Hawes | HITIQ |
| | T. Ide | BRG Sports (Equity holder in Riddell parent BRG Sports) |

| | | |
|---|---|---|
| | T. Laudenbach | HITIQ |
| | J. Mihalik | Senaptec, Inc - Co-Founder, Equity stakeholder |
| | M. Shogren | Prevent Biometrics |
| | J. Stitzel | Elemance, LLC – ownership interest |
| **Receipt of equip., materials, drugs, medical writing, gifts or other services** | B. Jones | I have received equipment from Biocore-FRI, HitIQ, Prevent and ORB to undertake a validity study. All costs associated with the study were funded by Leeds Beckett University. |
| | G. Siegmund | MIPS (equipment and advice for the construction of a free fall drop tower used for helmet testing.) |
| **Employment/salary from relevant entity** | A. Bartsch | Prevent Biometrics |
| | J. Chu | Simbex |
| | J. Crandall | Biocore LLC – Owner |
| | E. Falvey | World Rugby |
| | J. Funk | Biocore LLC |
| | L. Gabler | Biocore LLC |
| | D. Goodger | Prevent Biometrics |
| | R. Greenwald | Simbex |
| | D. Hawes | HITIQ |
| | K. Harmon | Pac-12 Research Development Director |

| | | |
|---|---|---|
| | T. Ide | Riddell |
| | T. Laudenbach | HITIQ |
| | M. Shogren | Prevent Biometrics |
| | G. Siegmund | MEA Forensic Engineering and Scientists |
| | C. Willmott | Australian Football League |
| | C. Withnall | Biokinetics and Associates, Ltd. |
| **Other** | A. Bartsch | Immediate family members own stock in Prevent Biometrics |
| | S. Broglio | Proceeds from Biomechanics of Injury (3rd edition, Human Kinetics) – book |
| | R. Kraft | BrainSim Technologies Inc. |
